# 5.5 GHz film bulk acoustic wave filters using thin film transfer process for WLAN applications

**DOI:** 10.1038/s41378-024-00820-3

**Published:** 2024-11-25

**Authors:** Tingting Yang, Chao Gao, Yaxin Wang, Binghui Lin, Yupeng Zheng, Yan Liu, Cheng Lei, Chengliang Sun, Yao Cai

**Affiliations:** 1 The Institute of Technological Sciences, Hubei Key Laboratory of Electronic Manufacturing and Packaging Integration, Wuhan University, 430072 Wuhan, China; 2 Hubei Yangtze Memory Laboratories, 430205 Wuhan, China; 3 School of Microelectronics, Wuhan University, 430072 Wuhan, China

**Keywords:** Electronic devices, Nanoscale devices

## Abstract

Wireless local area network (WLAN) has gained widespread application as a convenient network access method, demanding higher network efficiency, stability, and responsiveness. High-performance filters are crucial components to meet these needs. Film bulk acoustic resonators (FBARs) are ideal for constructing these filters due to their high-quality factor (*Q*) and low loss. In conventional air-gap type FBAR, aluminum nitride (AlN) is deposited on the sacrificial layer with poor crystallinity. Additionally, FBARs with single-crystal AlN have high internal stress and complicated fabrication process. These limit the development of FBARs to higher frequencies above 5 GHz. This paper presents the design and fabrication of FBARs and filters for WLAN applications, combining the high electromechanical coupling coefficient (${K}_{{\rm{t}}}^{2}$) of Al_0.8_Sc_0.2_N film with the advantages of the thin film transfer process. An AlN seed layer and 280 nm-thick Al_0.8_Sc_0.2_N are deposited on a Si substrate via physical vapor deposition (PVD), achieving a full width at half maximum (FWHM) of 2.1°. The ultra-thin film is then transferred to another Si substrate by wafer bonding, flipping, and Si removal. Integrating conventional manufacturing processes, an FBAR with a resonant frequency reaching 5.5 GHz is fabricated, demonstrating a large effective electromechanical coupling coefficient (${{k}}_{{\rm{eff}}}^{2}$) of 13.8% and an excellent figure of merit (FOM) of 85. A lattice-type filter based on these FBARs is then developed for the Wi-Fi UNII-2 band, featuring a center frequency of 5.5 GHz and a −3 dB bandwidth of 306 MHz, supporting high data rates and large throughputs in WLAN applications.

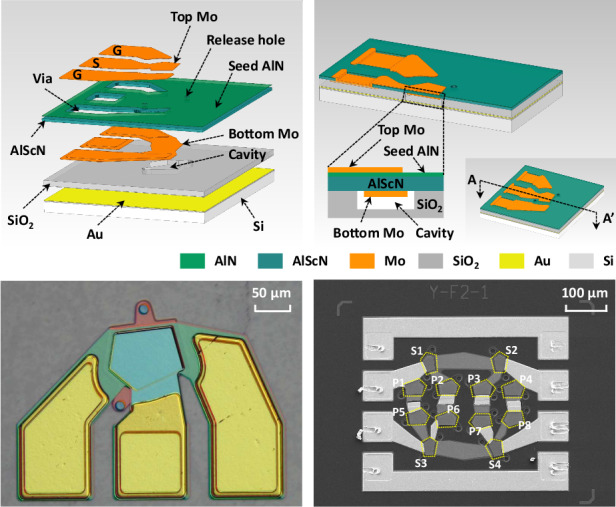

## Introduction

A wireless local area network (WLAN) is a system that uses wireless technology to facilitate data communication between devices within a limited geographic area. As the demand for higher data rates, greater network capacity, and lower latency continues to grow in enterprise networks, public hotspots, and smart cities, WLAN technology standards are evolving to meet these expanding application requirements^1^. Wi-Fi 6 (IEEE 802.11ax), as the latest WLAN standard, supports both 2.4 and 5 GHz bands. Within the 5 GHz band, there are subdivisions such as UNII-1 (5.150–5.250 GHz), UNII-2 (5.250–5.725 GHz), and UNII-3 (5.725–5.850 GHz)^2,3^. This complex channel allocation and management necessitate that the filters be precise in selecting and isolating frequencies, ensuring the fidelity and integrity of communications within these dense spectrums. The high-quality factor (*Q*) of film bulk acoustic resonator (FBAR) provides filters with highly accurate frequency selectivity, while its low insertion loss effectively reduces energy loss^4,5^. Consequently, FBARs are considered ideal choices for filters in high-frequency WLAN applications. The resonant frequency of FBARs is determined by the thickness of the piezoelectric film, with thinner piezoelectric layers promoting higher frequencies^6^. However, the reduction in thickness leads to an increase in defects and irregularities during deposition, resulting in significant degradation of crystal quality. These factors directly affect FBAR performance, impacting insertion loss (IL), effective electromechanical coupling coefficient (${{k}}_{{\rm{eff}}}^{2}$), and *Q* factor. Therefore, the fabrication of high-quality piezoelectric films is critical for the development of high-frequency FBARs. Currently, Aluminum nitride (AlN) and scandium-doped aluminum nitride (AlScN) are commonly used piezoelectric materials for FBARs due to their high longitudinal acoustic velocity and compatibility with complementary metal-oxide-semiconductor (CMOS)^7–12^. Doping Sc into AlN has been shown to enhance its piezoelectric properties^13–16^. Although manufacturing processes for AlN and AlScN-based FBARs are well-established for frequencies below 5 GHz, these challenges become more pronounced at higher frequencies.

In traditional air-gap FBAR utilizing AlN or AlScN as piezoelectric film, there are two common methods for forming the cavity. One method involves deep silicon etching of a thick silicon (Si) substrate (thickness approximately 500–725 µm)^17,18^. The other method involves pre-etching the cavity and filling it with a sacrificial material, typically SiO_2_ or amorphous silicon (a-Si), which is later etched away^19,20^. The first method requires the cavity to have a high aspect ratio, posing stringent demands on the Si etching process. Moreover, AlN is usually grown on Mo electrodes. The second method involves chemical-mechanical polishing (CMP) to planarize the surface of the substrate filled with the sacrificial layer before depositing the piezoelectric film. However, this process results in an uneven substrate surface due to CMP, increasing surface roughness^21^. Additionally, due to the crystallographic properties of SiO_2_ (or a-Si), the AlN film in the resonant region is more likely to form an amorphous or polycrystalline structure. Many studies have indicated that the quality of AlN thin films deposited on Si substrates is superior to those deposited on Mo electrodes or Mo/SiO_2_/Si substrates^19–21^. In this work, the piezoelectric film is directly deposited onto a Si substrate, which avoids the crystal quality degradation that occurs when AlN or AlScN piezoelectric films are deposited on sacrificial layers or Mo electrodes in the traditional FBAR process.

On the other hand, some researchers have turned to single-crystal AlN thin films for FBAR manufacturing to enhance film quality and improve the *Q* factor^22–25^. However, the production of single-crystal AlN is complex and expensive, often resulting in high internal stress due to lattice mismatch with substrates like sapphire or SiC^26^. Additionally, the high mechanical strength of these substrates makes it difficult to precisely remove without compromising the integrity of the functional AlN layer. This challenge is particularly significant for ultra-thin films, where even minor defects can severely impact performance. Thus, the thin-film transfer process was developed^27–29^. This method transfers piezoelectric film from the epitaxial substrate to a more suitable target substrate, ensuring both film quality and structural integrity while avoiding the challenges associated with traditional substrate etching methods. Current research on thin-film transfer processes has primarily focused on single-crystal AlN films. However, the limited electromechanical coupling coefficient (${K}_{{\rm{t}}}^{2}$) of AlN restricts its ability to provide the broader bandwidth required by WLAN.

In this work, we combined the high electromechanical coupling coefficient of Al_0.8_Sc_0.2_N thin film with the advantages of the thin film transfer process to fabricate FBARs and filters for the Wi-Fi UNII-2 band. The fabrication process begins with the deposition of an AlN seed layer and an Al_0.8_Sc_0.2_N on a Si substrate via PVD. The Al_0.8_Sc_0.2_N film, with a thickness of 280 nm, is subsequently characterized, exhibiting a full width at half maximum (FWHM) of 2.1° and a good *c*-axis orientation. Subsequently, the ultra-thin films are transferred to another Si substrate by wafer bonding, flipping, and Si removal. Combined with standard manufacturing processes, the FBAR is successfully fabricated, achieving a resonant frequency of up to 5.5 GHz. The fabricated FBAR demonstrated a maximum quality factor (*Q*_max_) of 619, a large effective electromechanical coupling coefficient (${{k}}_{{\rm{eff}}}^{2}$) of 13.8%, and an excellent figure of merit (FOM) (${Q}_{\max }\times {{\rm{k}}}_{{\rm{eff}}}^{2}$) of 85. A lattice-type filter designed for the Wi-Fi UNII-2 band is further developed. The fabricated filter operates with a center frequency of 5.5 GHz and a − 3 dB bandwidth of 306 MHz, spanning from 5.394 GHz to 5.700 GHz. This work demonstrates the feasibility of integrating Al_0.8_Sc_0.2_N thin films with the thin-film transfer process, achieving broader bandwidth in the fabricated filters. It expands the scope of materials for thin-film transfer beyond traditional single-crystal AlN, enabling the deposition of AlN films doped with varying Sc concentrations. This advancement paves the way for designing high-performance FBAR devices and allows for more flexibility in selecting materials tailored to specific application requirements.

### Design and fabrication

**Fig. 1:**
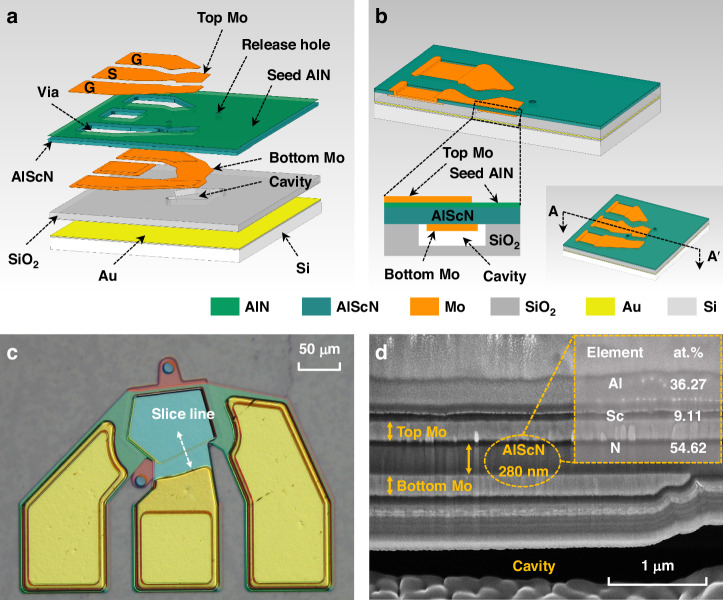
Al_0.8_Sc_0.2_N-based FBAR. **a** The exploded view of the FBAR, showing the stacking of various material layers and structural details. **b** The sectional view of the FBAR along the line A-A’. **c** The optical microscopy image of the fabricated FBAR. **d** The cross-sectional view of the fabricated FBAR along the slice line of **c** by SEM, and the atomic percentages of Al_0.8_Sc_0.2_N film characterized by EDS

The piezoelectric FBAR designed in this paper is depicted in Fig. 1. When an electric field is applied to the piezoelectric layer, it generates mechanical vibrations that propagate as bulk acoustic waves. Figure 1a provides an exploded view of the FBAR, showing the stacking of various material layers and structural details. The FBAR is produced on a 4-inch Si substrate, which possesses excellent mechanical stability and ease of processing. The functional layers, from top to bottom, are sequentially top molybdenum (Mo), AlN seed, Al_0.8_Sc_0.2_N, and bottom Mo, forming a sandwich structure. Connecting the bottom Mo to the top Ground–Signal–Ground (G–S–G) pad through a via facilitates efficient electrical signal transmission and reduces signal loss. In addition, the release holes in the FBAR provide a passage for the etching gases to react chemically with the sacrificial layer, creating a precisely defined cavity below the resonance region. Figure 1b presents the sectional view of the FBAR, illustrating the interrelationships among the functional layers and their connections to the substrate and pads.

Figure 1c, d depicts the surface morphology and cross-sectional view of the fabricated FBAR, characterized by optical microscopy and scanning electron microscopy (SEM). Specifically, Fig. 1d details the layering of the FBAR along the cross-section shown in the slice line of Fig. 1c, including the top electrode, AlN seed layer, the piezoelectric layer Al_0.8_Sc_0.2_N, and the bottom electrode. The elemental composition of Al_0.8_Sc_0.2_N is analyzed using an energy dispersive spectrometer (EDS). The atomic percentage (at.%) of the elements is calculated by quantifying the X-ray emission intensity of each element. The EDS results are depicted in the additional diagram in Fig. 1d, where the atomic percentages of Al and Sc are 36.27 at.% and 9.11 at.%, respectively. Therefore, the Sc doping concentration is calculated to be 20.07%, which is consistent with the expected doping content of the sample.

**Fig. 2:**
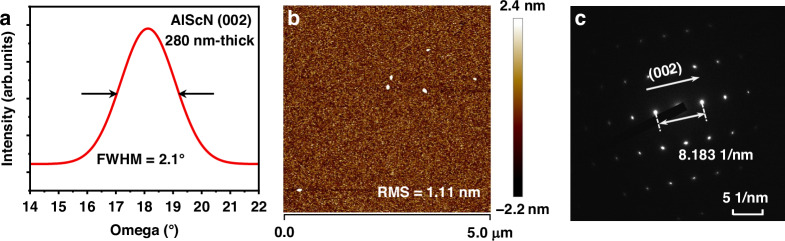
Characterization of Al_0.8_Sc_0.2_N piezoelectric film. **a** X-ray rocking curve of 280 nm-thick Al_0.8_Sc_0.2_N film, with a FWHM of 2.1°. **b** 5 μm × 5 μm AFM image of Al_0.8_Sc_0.2_N film, with a RMS of 1.11 nm. **c** SAED spectrum of Al_0.8_Sc_0.2_N

Figure 2a illustrates the X-ray rocking curve for the 280 nm-thick Al_0.8_Sc_0.2_N film, featuring a distinct peak that corresponds to the (002) crystallographic orientation with an FWHM of 2.1°. Figure 2b displays the atomic force microscope (AFM) morphology scan of the Al_0.8_Sc_0.2_N thin film, with the root mean square roughness (RMS) measured at 1.11 nm. It indicates that the deposition process is well-controlled, with minimal Sc precipitate grains present on the film surface. The selected area electron diffraction (SAED) pattern is shown in Fig. 2c. The distance between the two diffraction spots nearest to the central spot is 8.183 1/nm, which corresponds to the lattice plane spacing of 0.244 nm, matching the (002) crystal orientation. The enhanced intensity of these spots compared with other spots in the diffractogram again indicates higher crystallinity along the (002) orientation.

**Fig. 3:**
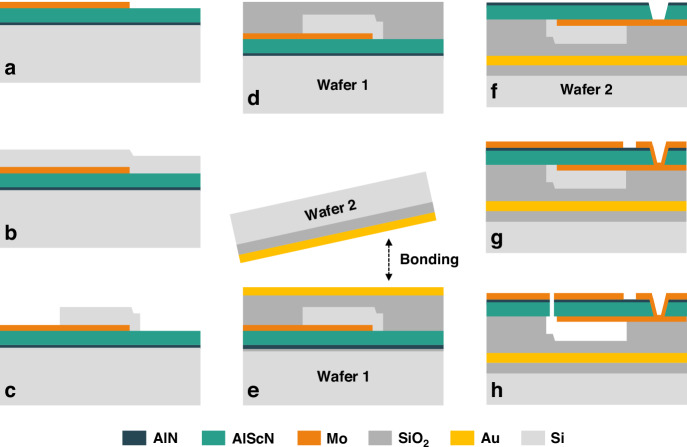
Fabrication process flow of the FBARs. **a** Sequential deposition of AlN seed layer, Al_0.8_Sc_0.2_N, and Mo stack, followed by patterning of Mo via ICP etching to form the bottom electrode. **b**, **c** Deposition and patterning of the a-Si sacrificial layer to create a predetermined cavity structure. **d** Deposition of SiO_2_ support layer and polishing the surface by CMP. **e** The film transfer from Wafer 1 to Wafer 2, including wafer bonding through the Au–Au interface, followed by removal of the Si substrate on Wafer 1 and global inversion. **f** Etching AlN and Al_0.8_Sc_0.2_N layers by ICP to form vias. **g** Deposition and patterning of the top Mo. **h** Etching the release holes by ICP and forming the cavities by chemical reaction

The fabrication process of the FBARs begins with the deposition of the AlN seed layer onto a Si substrate to improve the uniformity and crystal quality of the subsequent 280 nm-thick Al_0.8_Sc_0.2_N piezoelectric film. Following this, the Mo layer is deposited and patterned via Inductively Coupled Plasma (ICP) etching to form the bottom electrode, as shown in Fig. 3a. And a 1.5 μm-thick a-Si layer is deposited as a sacrificial layer, depicted in Fig. 3b, c. Then, through deep silicon etching, this layer is etched into a predetermined cavity structure. Following this, a 5 μm-thick layer of undoped SiO_2_ is deposited using plasma-enhanced chemical vapor deposition (PECVD). This layer serves as both a peripheral support for the cavity and as an insulating layer for the subsequent Au layer. CMP is then employed to polish the surface to make it uniform and smooth, as shown in Fig. 3d.

**Fig. 4:**
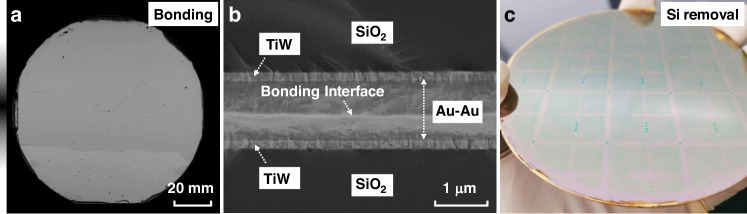
Characterization of wafer bonding. **a** SAM image of the wafer bonded at 350 °C. **b** The cross-sectional view of the Au–Au bonding interface by SEM. **c** The wafer surface after Si removal

The subsequent stage is wafer bonding, shown in Fig. 3e, which is a critical step in the ultra-thin film transfer. Inadequate bonding can lead to the fracturing of the film during transfer. In this study, metal thermocompression bonding is adopted, which is combined by direct diffusion of intermetallic atoms and forms a bonding interface with high stability^30^. Aurum (Au) is widely used in this method because it can be bonded at relatively low temperatures (300–400 °C)^31,32^. A 200 nm-thick Titanium–Tungsten (TiW) adhesion layer followed by a 500 nm-thick Au are sequentially sputtered onto the surface of the cap wafer (termed Wafer 2) and the original wafer (termed Wafer 1). Subsequently, the two wafers are bonded through the Au–Au interface at a pressure of 100,000 N and a temperature of 350 °C for 1 h. Scanning acoustic microscopy (SAM) is used to inspect the defects, as shown in Fig. 4a, where a few defects are observed and the entire wafer area is bonded. Due to its high plasticity and ductility, Au is able to compensate for the micro-roughness on the contact surface during the thermocompression bonding, resulting in a more uniform bonding interface. The cross-sectional view is further observed by SEM, as shown in Fig. 4b, where the Au–Au bonding interface is nearly devoid of voids and the entire surface is uniformly bonded. Subsequently, the assembly is inverted to remove the Si substrate of Wafer 1. Initially, the Si is thinned to approximately 100 μm using dry etching. The remaining Si is then completely removed through wet etching with a mixed solution of HNO_3_ and HF, thereby exposing the underlying piezoelectric thin film. To prevent the Si on the opposite side from reacting during the wet etching process, the wafer is sealed to the sapphire substrate using paraffin wax. As shown in Fig. 4c, the wafer surface after Si removal remains intact with little device damage, successfully completing the ultra-thin film transfer.

As illustrated in Fig. 3f, the AlN and Al_0.8_Sc_0.2_N layers are then etched using ICP to create vias. A Mo layer is subsequently deposited and patterned to constitute the top electrode, shown in Fig. 3g. Finally, the cavity formation is achieved by etching release holes and removing the sacrificial a-Si layer through a chemical reaction with Xenon Difluoride (XeF_2_), as illustrated in Fig. 3h, thereby completing the fabrication of the FBARs.

## Results and discussion

**Fig. 5:**
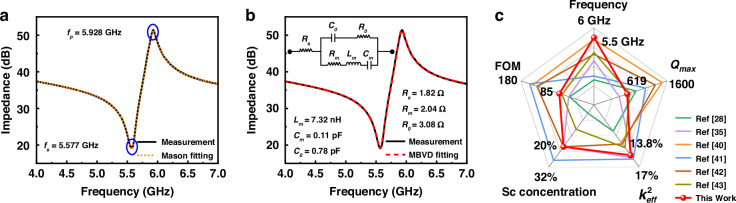
Measurement and analysis of the fabricated FBARs. **a** The fitted curve from the Mason model and the measured impedance curve. **b** Measured and MBVD fitted plot for the FBAR. **c** Comparison of the performance of AlScN-based FBARs with others research

The frequency response curves of the FBARs are measured by a Cascade Microtech GSG probe station (Cascade, USA) and a network analyzer (N5222B, Agilent Technology). The layer thicknesses used in the Mason model are determined from accurate measurements of the stacked layer cross-section by SEM. Subsequently, the acoustic impedance *Z* and the longitudinal acoustic velocity *v*_*a*_ are fine-tuned to align with the anti-resonance frequency *f*_*p*_. Similarly, the ${K}_{t}^{2}$ of piezoelectric film is calibrated to match the resonance frequency *f*_*s*_. The resulting fitted curves are shown in Fig. 5a. The other parameters of the piezoelectric film can then be calculated according to the following equation^33^.

1 $$\rho =\frac{Z}{{v}_{a}}$$

2 $${c}_{33}^{D}=\rho {v}_{a}^{2}$$

3 $${e}_{33}=\sqrt{{K}_{t}^{2}{c}_{33}^{D}{\varepsilon }_{r}{\varepsilon }_{0}}$$

4 $${C}_{33}^{E}={c}_{33}^{D}-\frac{{e}_{33}^{2}}{{\varepsilon }_{r}{\varepsilon }_{0}}$$

where *ρ* is the density, ${c}_{33}^{D}$ is the stiffened elastic constant, ${C}_{33}^{E}$ is the elasticity constant, *e*_33_ is the piezoelectric stress constant, *ε*_*r*_ is relative permittivity, and *ε*_*0*_ is the vacuum dielectric constant (${\varepsilon }_{0}=8.854\times {10}^{-12}$ F/m). Table 1 provides a comparison of the material parameters for Al_0.8_Sc_0.2_N films obtained in this work with those reported in other research. The electromechanical coupling coefficient ${K}_{{\rm{t}}}^{2}$ of the Al_0.8_Sc_0.2_N film reaches 16.3%, which represents a significant enhancement in performance compared to pure AlN films (6%)^34^. And the ${K}_{{\rm{t}}}^{2}$ is superior compared to the previously reported parameters of Al_0.8_Sc_0.2_N films with the same Sc doping concentration^35,36^, which may be attributed to improvements in film quality. It allows for wider bandwidths in resonators and filters. In addition, the Al_0.8_Sc_0.2_N film exhibits a ${e}_{33}$ of 2.38 C/m^2^, which is higher than that of AlN films, typically 1.55 C/m^2 ^^37^. This increased piezoelectric response enhances the efficiency of energy conversion.

**Table 1:** Comparison of material parameters for AlN and Al_0.8_Sc_0.2_N film

	Material	${K}_{t}^{2}$ (%)	${\varepsilon }_{r}$	*ρ* (kg/m^3^)	${C}_{33}^{E}$ (GPa)	*V*_*a*_ (m/s)	*e*_*33*_ (C/m^2^)
^44^	AlN	6.5	–	–	435	–	1.55
^34^	AlN	6	9.5	3260	395	–	1.55
^36^	Al_0.8_Sc_0.2_N	14.5	12.2	3500	287	–	–
^35^	Al_0.8_Sc_0.2_N	12.4	13.42	3560	258	9089	2.08
This work	Al_0.8_Sc_0.2_N	16.3	13.76	3550	239	8978	2.38

Furthermore, the modified Butterworth–Van Dyke (MBVD) equivalent circuit model for the FBAR has been formulated. This model is composed of two principal branches: the first encompasses the static capacitance (*C*_0_), electrode resistance (*R*_*s*_), and dielectric loss (*R*_0_), while the second comprises the motional resistance (*R*_*m*_), motional inductance (*L*_*m*_), and motional capacitance (*C*_*m*_). The MBVD model and the fitting results are shown in Fig. 5b, with the extracted static capacitance ${C}_{0}$ = 0.78 pF. The reference resonator has an area of 1500 μm^2^, and using the theoretical formula ${C}_{0}={\varepsilon }_{{\rm{r}}}{\varepsilon }_{0}A/d$, the estimated is 0.65 pF. The discrepancy between the estimated and fitted values arises because the theoretical calculation assumes ideal geometric and material conditions, which may overlook real factors such as fabrication tolerances, parasitic effects, and edge effects. And at high frequencies, the necessity for thin electrodes may result in insufficient adhesion between the electrodes and the piezoelectric material, leading to increased interfacial friction and mechanical damping. Additionally, thinner electrodes possess higher intrinsic resistance, resulting in additional energy loss. Consequently, the *R*_*s*_, *R*_*m*_, *R*_0_ in the MBVD model are elevated, leading to a decrease in the quality factor series quality factor *Q*_*s*._

From the measured impedance curve, the series resonant frequency *f*_*s*_ = 5.577 GHz and parallel resonant frequency *f*_*p*_ = 5.928 GHz of the FBAR can also be obtained. ${{k}}_{\rm{eff}}^{2}$ is calculated as 13.8% according to formula (5)^38^.

5 $${{k}}_{\rm{eff}}^{2}=\frac{{\pi }^{2}}{4}\cdot \frac{{f}_{s}\cdot ({f}_{p}-{f}_{s})}{{f}_{p}^{2}}$$

And *Q* is calculated using the Bode method according to formula (6)^39^,

6 $$Q=\omega \cdot \frac{|{S}_{11}|{\rm{group}}\_{\rm{delay}}({S}_{11})}{1-{|{S}_{11}|}^{2}}$$

where *ω* is the angular resonant frequency ($\omega =2\pi {\rm{f}}$) and *S*_11_ is the reflection coefficient parameter. In this study, the FBAR exhibits a series quality factor *Q*_*s*_ of 116, a parallel quality factor *Q*_*p*_ of 418, and a maximum quality factor *Q*_max_ of 619.

Figure 5c compares the performance metrics of the AlScN-based FBARs fabricated in this study with those reported in other research works^28,35,40–43^, focusing on frequency, *Q*_max_, ${{k}}_{\rm{eff}}^{2}$, and FOM. The FBAR in this study achieves an ultra-high operational frequency of 5.5 GHz, with a ${{k}}_{\rm{eff}}^{2}$ of 13.8% using Al_0.8_Sc_0.2_N film. These results represent a significant improvement over the FBAR based on AlN films, which exhibit a ${{k}}_{\rm{eff}}^{2}$ of 7.2%^30^. In the development of high-performance FBAR-based filters, it is critical to prioritize the overall functionality of the device while also considering specific performance metrics. Here, the FBAR is designed for the Wi-Fi UNII-2 band, and the results indicate that the frequency, bandwidth, and *Q* meet the requirements.

**Fig. 6:**
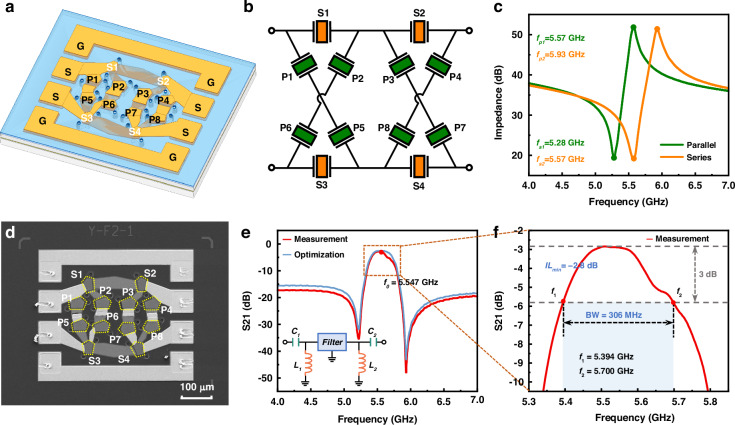
Experimental results of fabricated FBARs filter. **a** Schematic diagram of a two-order lattice-type filter. **b** Schematic circuit design of the lattice-type filter. **c** The frequency response curves of the series and parallel resonators. **d** The surface view of the fabricated filter taken by SEM. **e** Measured the S21 transmission response curve of the fabricated filter and the simulation curve with the added matching circuit. **f** A detailed zoomed-in view of the measurement curve from 5.30 to 5.85 GHz

Furthermore, a two-order lattice-type filter for the Wi-Fi UNII-2 band is developed, as depicted in Fig. 6a. The filter consists of four series resonators (S1–S4) and eight parallel resonators (P1–P8). The area of each resonator and the interconnections between them are optimized in the design. The series resonators have the same area of 750 μm^2^. And considering the reduced power capacity issue due to the small area of resonators at high frequencies, an area multiplication method is used on the parallel path. This method involves replacing a small area resonator with two larger resonators connected in series, each with an area double that of the original small resonator. Consequently, the areas of the parallel resonators are 1060 μm^2^. The estimated *C*_*0*_ for the series and parallel resonators are 0.33 pF and 0.46 pF, respectively. However, coupling effects between resonators can significantly influence their resonant frequencies and capacitance values. The interconnection of multiple resonators introduces mutual inductance, parasitic capacitance, and complex impedance interactions, which can lead to deviations between the theoretical estimates and actual values. Externally, a Ground–Signal–Signal–Ground (GSSG) pad layout is employed to enhance the protection against external electromagnetic interference, especially in high-frequency applications. Its circuit schematic design is depicted in Fig. 6b.

Figure 6c illustrates the frequency response curves of the parallel and series resonators, detailing their resonant (*f*_*s*1_, *f*_*s*2_) and anti-resonant frequencies (*f*_*p*1_, *f*_*p*2_). Table 2 lists the key parameters of the resonators used in constructing the filter. The frequency of the parallel resonator is adjusted by adding a Mo mass loading on the top electrode, in order to match *f*_*p*1_ with *f*_*s*2_. Proper frequency matching ensures that the center frequency of the filter is accurately set, providing the desired operational band. Meanwhile, *f*_*s*1_ and *f*_*p*2_ in this configuration define the left and right transmission zeros of the filter, respectively. This is important for preventing interference from adjacent channels and ensuring clear signal transmission within the desired band.

**Table 2:** The key parameters of the series and parallel resonators used in the filter

Resonator	*A* (μm^2^)	*f*_*s*_ (GHz)	*f*_*p*_ (GHz)	${\boldsymbol{k}}_{\mathbf{eff}}^{\boldsymbol{2}}\,$ (%)	Estimated *C*_0_ (pF)
Series (S1–S4)	750	5.57	5.93	13.8	0.33
Parallel (P1–P8)	1060	5.28	5.57	12.6	0.46

Figure 6d shows the surface view of the fabricated filter taken by SEM. The measured transmission response of the fabricated filter is shown in Fig. 6e, and Fig. 6f provides a detailed zoomed-in view of the measurement curve from 5.30 GHz to 5.85 GHz. Table 3 provides a comparison of the performance metrics of AlScN-based filters from this study with those reported in other research works. The filter fabricated in this work, utilizing a 280 nm ultra-thin Al_0.8_Sc_0.2_N film, achieved a center frequency of 5.5 GHz and a bandwidth of 306 MHz. When compared to devices using the same Sc concentration of Al_0.8_Sc_0.2_N film^35,42^, the bandwidth is nearly equivalent, and it even exceeds that of devices using a higher Sc concentration, such as Al_0.72_Sc_0.28_N, which achieved a bandwidth of 216 MHz^41^. The minimum insertion loss (*IL*_min_) is −2.8 dB. Furthermore, the filter exhibits a near-band rejection of greater than −30 dB and a far-end rejection of greater than −17 dB, ensuring minimal interference from adjacent frequency bands and improved signal clarity. These results demonstrate the feasibility of applying AlScN in thin-film transfer processes and highlight the potential of the fabricated filters for high-frequency applications, particularly in the Wi-Fi UNII-2 band.

**Table 3:** Comparison of the performance of AlScN-based filters with other research

	Materials	Method	Thickness	Substrate	*f*_0_ (GHz)	*IL*_min_ (dB)	BW/−3 dB	Rejection (dB)
^28^	AlN	MOCVD	650 nm	SiC	3.4	1.6	160	>31
^41^	Al_0.72_Sc_0.28_N	PVD	678 nm	Si	3.5	0.87	216	>5
^45^	AlN	PVD	500 nm	Mo	4.2	3.2	83	>30
^35^	Al_0.8_Sc_0.2_N	PVD	503 nm	Si	4.24	1.88	215	>32
^42^	Al_0.8_Sc_0.2_N	MOCVD	400 nm	Si	4.8	0.78	390	>20
^43^	Al_0.904_Sc_0.096_N	PVD	401 nm	Si	4.85	0.93	240	>25
^46^	AlN	MOCVD	600 nm	Si	5.24	2	210 (−4 dB)	>28.5
^47^	AlN	MOCVD	500 nm	SiC	5.24	2.82	151 (−4 dB)	>38
^48^	AlN	NA	NA	NA	6.02	2	160	>40
^42^	Al_0.75_Sc_0.25_N	MOCVD	NA	Si	7.2	2.2	914	>15
This Work	Al_0.8_Sc_0.2_N	PVD	280 nm	Si	5.5	2.8	306	>17

Furthermore, the matching circuit of the filter has been optimized, as illustrated in Fig. 6e, consisting of capacitors (*C*_1_, *C*_2_) and inductors (*L*_1_, *L*_2_), where *C*_1_ = 62.9 pF, *C*_2_ = 85.4 pF, *L*_1_ = 13.3 nH, and *L*_2_ = 9.8 nH. By incorporating the circuit, the flatness of the filter’s passband response has been improved, achieving lower insertion loss and further enhancing the efficiency of signal transmission, which has a positive effect on the performance enhancement of the filter.

## Conclusion

In conclusion, this study designed and implemented a high-frequency FBAR and filter with 280 nm-thick Al_0.8_Sc_0.2_N as the piezoelectric film. The microstructure, crystal structure, and elemental composition of the Al_0.8_Sc_0.2_N film deposited on the Si substrate are characterized. The results show that Al_0.8_Sc_0.2_N has a good *c*-axis orientation, and the Sc doping concentration is well-matched to the design. Combining the thin film transfer process, the FBAR is successfully fabricated, achieving a resonant frequency of up to 5.5 GHz. Additionally, the fabricated FBAR demonstrated a *Q*_max_ of 619, a large ${k}_{\rm{eff}}^{2}$ of 13.8%, and an excellent FOM of 85. The Mason and MBVD models are combined with experimental measurements to accurately characterize the material parameters of the Al_0.8_Sc_0.2_N film and the electrical parameters of the FBAR. Furthermore, a lattice-type filter is fabricated with a center frequency of 5.5 GHz, a 3 dB bandwidth of 306 MHz, and the *IL*_min_ of −2.8 dB. The matching circuit is designed to optimize the filter passband performance. This filter is capable of supporting higher data rates and large throughputs in the Wi-Fi UNII-2 band. This work presents a novel manufacturing process for FBAR devices, and the fabricated devices demonstrate promising application potential in WLAN.
